# Ganglioside GM1 promotes contact inhibition of growth by regulating the localization of epidermal growth factor receptor from glycosphingolipid‐enriched microdomain to caveolae

**DOI:** 10.1111/cpr.12639

**Published:** 2019-05-24

**Authors:** Dinghao Zhuo, Feng Guan

**Affiliations:** ^1^ Key Laboratory of Carbohydrate Chemistry and Biotechnology, Ministry of Education, School of Biotechnology Jiangnan University Wuxi China; ^2^ Provincial Key Laboratory of Biotechnology, Joint International Research Laboratory of Glycobiology and Medicinal Chemistry, College of Life Science Northwest University Xi'an China

**Keywords:** caveolae, contact Inhibition of Growth, Epidermal Growth Factor Receptor, ganglioside GM1, glycolipids‐enriched Microdomain

## Abstract

**Objectives:**

Accumulating data show that gangliosides are involved in regulation of cell proliferation. Specific changes in gangliosides expression associated with growth density of cells have been documented in several cell lines. However, the function and the potential mechanism of ganglioside GM1 in contact inhibition of growth are not clear.

**Materials and Methods:**

EdU incorporation assay and western blot were applied to detect the contact inhibition of growth in human mammary epithelial cells. GM1 manipulation of cell proliferation and epidermal growth factor receptor (EGFR) activation was investigated by immunoprecipitation, OptiPrep density gradient centrifugation and immunofluorescence. The function of GM1 on contact inhibition of growth was further studied by using GM1 stably knockdown and overexpression cells.

**Results:**

MCF‐10A, MCF‐7 and MDA‐MB‐231 cells showed contact inhibition of growth in high‐density condition. Exogenous addition of GM1 to high‐density cells clearly inhibited cell growth and deactivated EGFR signalling. Compared to normal‐density cells, distribution of EGFR in high‐density cells was decreased in glycosphingolipid‐enriched microdomain (GEM), but more concentrated in caveolae, and incubation with GM1 obviously promoted this translocation. Furthermore, the cell growth and EGFR activation were increased in GM1 stably knockdown cells and decreased in GM1 stably overexpression cells when cultured in high density.

**Conclusions:**

Our results demonstrated that GM1 suppressed EGFR signalling and promoted contact inhibition of growth by changing the localization of EGFR from GEM to caveolae.

## 1 INTRODUCTION

Gangliosides, a sialic acid‐containing subtype of glycosphingolipids (GSLs), are typically anchored in the outer leaflet of the plasma membranes and mainly clustered in glycosphingolipid‐enriched microdomain (GEM), in mammals cells.^1^ Gangliosides have displayed many essential biological roles such as modulating cell growth, cell motility, cell adhesion and receptor recognitions.^2^ Especially, gangliosides are found to interact with many transmembrane growth factor receptors, including the epidermal growth factor receptor (EGFR), the platelet‐derived growth factor receptor (PDGFR) and the nerve growth factor receptor (NGFR).^3^ For example, exogenous addition of ganglioside GM3 inhibited tyrosine kinase activity of the EGFR in hepatoma cells.^4^ Accumulation of ganglioside GD2 enhanced proliferation and tumorigenicity of MDA‐MB‐231 breast cancer cells through activation of hepatocyte growth factor receptor (HGFR, also named c‐Met).^5^ Another ganglioside GM1, a receptor for cholera toxin, functions as a specific endogenous activator of NGFR in rat pheochromocytoma PC12 cells, and these enhanced effects seem to be due to the interaction of GM1 with Trk.^6^

In multicellular organisms, the strict size control of tissues and organs during development is the most fundamental to support multicellularity.^7^ In vitro, the proliferation of cultured normal cells is ceased when the cells come into contact and a confluent monolayer is formed, a phenomenon termed density‐dependent inhibition of cell growth or contact inhibition.^8^ In the process of contact inhibition, the tumour suppressor Merlin plays the essential role by modulating EGFR and its downstream signalling proteins MAPK (Erk1/2).^9^ And the proliferative response to growth factors is obviously reduced in high‐density cells.^10^ It has been found that expression of the ganglioside GD3 and the GSL Gb3 was increased in contact‐inhibited cells and knocked down of their synthase significantly suppressed contact inhibition,^11^ which suggests GSLs may have a role in cell density‐dependent regulation of cell growth. However, the molecular mechanisms underlying GSLs regulate contact inhibition have poorly been clarified.

Plasma membranes are structurally heterogenous and compartmental, and presence of a particular type of membrane microdomain known as lipid rafts, which consisting of dynamic assemblies of sterols and sphingolipids.^12^ Lipid rafts are involved in many cellular processes and in related signal transductions.^13^ GEM and caveolae are two main types of lipid rafts, and both are related to many receptors stimulation, such as EGFR, PDGFR, TrkA and so on.^14^ But GEM and caveolae showed different effects on these receptors, and changes in the receptors distribution in these two domains may lead to abnormal receptors function.^15^ As a functional component of GEM, GM1 may have a role in regulation of receptors activation and contact inhibition of growth.

In this study, we found that the growth of human mammary epithelial MCF‐10A, MCF‐7 and MDA‐MB‐231 cells was inhibited in high‐density condition, and the expression of GM1 was increased in contact‐inhibited cells. In addition, exogenous addition of GM1 promoted contact inhibition of growth and inhibited activation of EGFR signalling in high‐density cells. Furthermore, the underlying mechanism of GM1 in regulation of EGFR activation and inhibition of cell growth was explored.

## 2 MATERIALS AND METHODS

### 2.1 Reagents

Dulbecco's modified Eagle's medium (DMEM) and penicillin/streptomycin were purchased from HyClone (Logan, UT, USA). Foetal bovine serum was from Biological Industries (Kibbutz Beit Haemek, Israel). DMEM/F12, horse serum, was obtained from Gibco (Life Technologies GmbH, Karlsruhe, Germany). Cholera toxin, Cholera Toxin B subunit (CTB), fluorescein isothiocyanate (FITC)‐conjugated CTB, recombinant human insulin, bovine serum albumin (BSA), 2‐(N‐morpholino)‐ethanesulfonic acid (Mes), n‐octyl‐β‐D‐glucopyranoside (OGP), OptiPrep and DAPI were purchased from Sigma‐Aldrich (St Louis, MO, USA). Recombinant human epidermal growth factor (EGF) was purchased from Peprotech (Rocky Hill, NJ, USA).

### 2.2 Cell line and cell culture

Human mammary epithelial cell line MCF‐10A and human breast adenocarcinoma cell lines MCF‐7 and MDA‐MB‐231 were purchased from the Cell Bank at the Chinese Academic of Science (Shanghai, China). MCF‐10A and derivative cells were cultured in DMEM/F12 supplemented with 5% horse serum, 20 ng/mL EGF, 10 μg/mL recombinant human insulin, 0.1 μg/mL cholera toxin, 0.5 mg/mL hydrocortisone and 1% penicillin/streptomycin. MCF‐7, MDA‐MB‐231 and derivative cells were cultured in DMEM supplemented with 10% foetal bovine serum and 1% penicillin/streptomycin. All cells were cultured at 37°C in humidified atmosphere of 5% CO2 in air. Normal‐density cells were prepared by seeding at 2 × 10^4^/cm^2^ and culturing for 2 days. High‐density cells were prepared by seeding at 1 × 10^5^/cm^2^ (MCF‐10A and MCF‐7) or 1.5 × 10^5^/cm^2^ (MDA‐MB‐231) and culturing for 2 days.

### 2.3 5‐ Ethynyl‐2′‐deoxyuridine (EdU) incorporation assay

EdU incorporation assay was performed with iClick™ EdU Andy Fluor 647 Flow Cytometry Assay Kit (GeneCopoeia, Rockville, MD, USA) according to the manufacturer's instructions. In brief, cells were incubated with 50 μmol/L EdU for 4 hours at 37°C, and the cells were fixed with 4% paraformaldehyde (PFA) and permeabilized with 0.3% Triton X‐100. iClick reaction cocktail was added to react with the EdU for 30 minutes, and cells were analysed by flow cytometry (ACEA Biosciences, San Diego, California, USA).

### 2.4 Western blot analysis

Cells were lysed in RIPA buffer (50 mmol/L Tris‐HCl, 150 mmol/L NaCl, 1% sodium deoxycholate, 1% Triton X‐100, 0.1% SDS and pH 7.4) supplemented with protease inhibitor cocktail (Selleck Chemicals, Houston, TX, USA) and phosphatase inhibitor cocktail (Sigma‐Aldrich). Proteins were subjected and separated on SDS‐PAGE gel and transferred to PVDF membranes (Millipore, Burlington, MA, USA). After blocking with non‐fat milk or BSA, membranes were incubated overnight with the following specific primary antibodies: EGFR, phospho‐EGFR (Y1068), ERK1/2, phospho‐ERK1/2 (T202/T204), phospho‐Merlin (S518) (CST, Danvers, MA, USA), β‐tubulin, GAPDH (Sigma‐Aldrich), Merlin, caveolin‐1 (Santa Cruz Biotechnology, Santa Cruz, CA, USA), B3GALT4, Na+/K + ATPase (Abcam, Cambridge Cambridgeshire, UK), flotillin‐1 and flotillin‐2 (BD Biosciences, Franklin Lakes, NJ, USA). The membranes were then incubated with appropriate HRP‐conjugated secondary antibodies. Signals were visualized using ECL solution (Vazyme, Nanjing, China) and detected with gel documentation system (Tanon, Shanghai, China).

### 2.5 GSL extraction and analysis

GSL extraction and analysis were performed as described previously.^16^ Briefly, cells were extracted twice with 2 mL of isopropanol/hexane/water (55:25:20) and the extraction was concentrated to dry under nitrogen stream. Phospholipids were hydrolysed in 2 mL of 0.1 mol/L NaOH in methanol at 40°C for 2 hours, followed by neutralization with 1 mol/L HCl. 2 mL of hexane was added, and the upper phase was removed. GSLs in the lower phase were dried and solubilized in 1 mL of distilled water. The solution was adsorbed on Bond Elut C18 columns (Agilent Technologies, Palo Alto, CA), washed with water and eluted with chloroform/methanol (2:1). GSLs eluted were analysed by high‐performance thin layer chromatography (HPTLC) silica gel (Millipore) and stained with orcinol in sulphuric acid.

### 2.6 Co‐immunoprecipitation (Co‐IP) assay

Cells were washed three times with cold PBS and lysed in IP buffer (50 mmol/L Tris‐HCl, 150 mmol/L NaCl, 1% Triton X‐100, 60 mmol/L OGP and pH 8.0) containing protease inhibitor cocktail. The supernatants were collected and immunoprecipitated with EGFR agarose‐conjugated antibody (Santa Cruz) overnight at 4°C with gently rotation, and the control group was incubated with normal rabbit IgG and Protein A/G PLUS‐Agarose (Santa Cruz). The immune complexes were collected by centrifugation (1000 g), and the precipitates were washed three times with cold PBS. The pellets were resuspended in 1 x electrophoresis sample buffer and boiled, and analysed with western blot.

### 2.7 Flow cytometry assay

Cells were digested and washed with PBS, fixed with 4% PFA and blocked with 1% BSA in PBS. Cells were pelleted, resuspended in PBS and incubated with CTB‐FITC in dark for 30 minutes. After rinsing with PBS, cells were analysed by flow cytometry.

### 2.8 Isolation of lipid raft and non‐raft membrane fractions

Lipid raft and non‐raft membranes were isolated using a modified successive detergent extraction.^17^ In brief, cells were washed in PBS, resuspended in buffer A (25 mmol/L Mes, 150 mmol/L NaCl and pH 6.5) and added with an equal volume of the same buffer supplemented with 2% Triton X‐100 and protease inhibitor cocktail. After 30 minutes of incubation on ice, lysates were centrifuged and supernatants were collected as non‐raft membrane fractions. Insoluble pellets were resuspended in buffer B (1% Triton X‐100, 10 mmol/L Tris, 0.5 mol/L NaCl, 60 mmol/L OGP and pH 7.6) for 30 minutes on ice. Debris was pelleted, and supernatants were collected as lipid raft membrane fractions.

### 2.9 Fractionation by density gradient centrifugation

Further separation of membrane fractions by OptiPrep density gradient was performed as described previously.^18^ In brief, one D150 mm (high‐density cells) or four D150 mm (normal‐density cells) plates of cells were washed and lysed in buffer C (20 mmol/L Tris‐HCl, 250 mmol/L sucrose and pH 7.8) containing 1 mmol/L CaCl_2_ and 1 mmol/L MgCl_2_, and protease inhibitor cocktail by passage through a 3‐inch 22‐gauge needle 20 times. Lysates were centrifuged, and the post‐nuclear supernatant was collected and transferred to a new tube. The pellet was homogenized again in buffer C, and the second post‐nuclear supernatant was collected. Two post‐nuclear supernatants were combined, mixed with equal volume of 50% OptiPrep and placed at the bottom of a 12 mL centrifuge tube. Equal volumes of 20%, 15%, 10% and 0% OptiPrep were in buffer C and were carefully overlaid above of the lysate (25% OptiPrep). The samples were centrifuged for 90 minutes at 52 000 g in an SW‐41 rotor (Beckman Coulter, Miami, FL, USA). Twelve 1 mL fractions were collected from the top of the tube, and equal volume aliquots of each fraction were subjected to western blot analysis.

### 2.10 Immunofluorescence staining

Cells were cultured on a glass bottom dish, fixed with PFA, blocked with BSA and incubated with specific primary antibodies for EGFR, caveolin‐1 (Abcam) and flotillin‐2 overnight. Cells were then incubated with Alexa fluorophore‐conjugated (−488, −647) secondary antibodies in dark for 1 hour. For GM1 staining, cells were incubated with CTB‐FITC in dark for 2 hours. Nuclei were counterstained with DAPI. Fluorescence images were obtained via a confocal microscopy (FluoView FV1000; Olympus, Tokyo, Japan).

### 2.11 RNA extraction and quantitative real‐time PCR

Total RNA was extracted and purified using an Ultrapure RNA Kit (CWBIO, Beijing, China) following the manufacturer's instructions. 1 μg of total RNA was used to synthesize the first‐strand cDNA using HiScript II Q RT SuperMix (Vazyme) with random primers. Real‐time PCR was performed in a BioRad CFX‐96 real‐time system (BioRad, Hercules, CA, USA) with AceQ qPCR SYBR Green Master Mix (Vazyme) according to the manufacturer's instructions. The upstream and downstream primers of target mRNA were described as follows: forward 5’‐GACGCTATTCTTGCTGGGAG‐3’ and reverse 5’‐TTAGGGTGAGGTTGCGGTAG‐3’ for B3GALT4; forward 5’‐ACCCACTCCTCCACCTTTG and reverse 5’‐CTCTTGTGCTCTTGCTGGG‐3’ for GAPDH. Relative expression of B3GALT4 was normalized to internal controls (GAPDH), and results were calculated with 2−∆∆Ct method.^19^

### 2.12 Lentiviral construction and viral infection

Full‐length of human B3GALT4 gene, which is responsible for GM1 synthesis, was cloned into the lentiviral vector pLVX (Addgene, Cambridge, MA, USA). For RNA interference, short hairpin RNAs (shRNA) with the complementary sequences of the target genes were cloned into the lentiviral vector pSicoR (Addgene). The target sequences for the shRNA were the B3GALT4 shRNA, 5’‐GACGGACGATGATGTGTAT‐3’, and a scrambled shRNA was used as a control. Cells were infected with lentivirus, and stable transfected cells were selected with puromycin.

### 2.13 Statistical analysis

All statistical analyses were carried out by GraphPad Prism version 7.0 software. Data sets between two groups were analysed using a two‐tailed Student's *t* test, and *P* < 0.05 was considered as statistical significance.

## 3 RESULTS

### 3.1 MCF‐10A, MCF‐7 and MDA‐MB‐231 cells showed contact inhibition of growth

**Figure 1:**
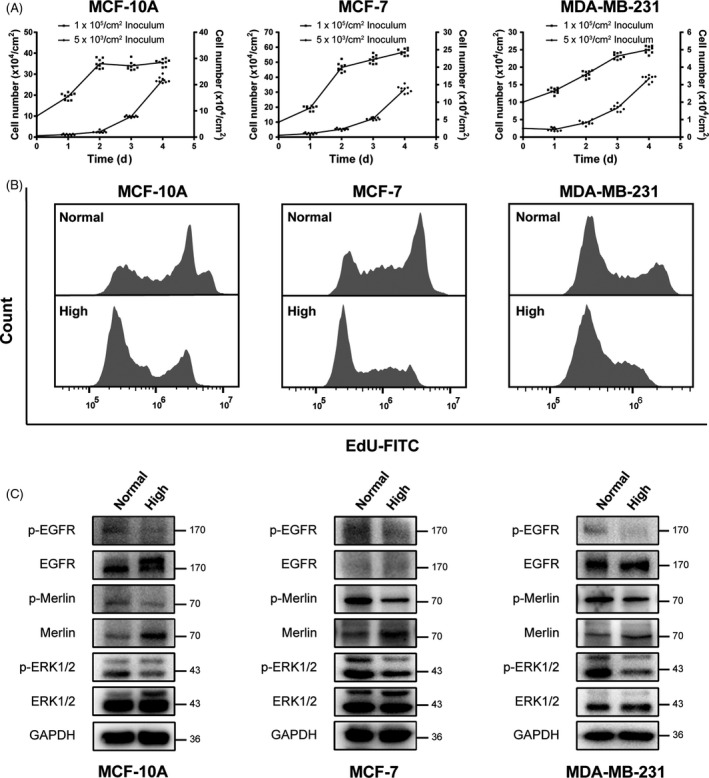
Contact inhibition of growth in human mammary epithelial cells. A, Cells were seeded at 5 × 10^3^/cm^2^ and 1 × 10^5^/cm^2^ and cultured for 4 d, and the medium was replaced with fresh medium after 2 d of culture. Cells were obtained by trypsin digestion and counted using an Automated Cell Counter. 1 × 10^5^/cm^2^ inoculum corresponded to the left y‐axis，and 5 × 10^3^/cm^2^ inoculum corresponded to the right y‐axis. B, Cells were seeded at normal (2 × 10^4^/cm^2^) and high (1 × 10^5^/cm^2^ for MCF‐10A and MCF‐7, 1.5 × 10^5^/cm^2^ for MDA‐MB‐231) density and cultured for 2 d. Cells were incubated with EdU followed by flow cytometry analysis. C, Phosphorylation levels of EGFR, ERK1/2 and Merlin in normal‐ and high‐density cells were analysed by western blot. GAPDH was used as loading control

To examine the contact inhibition of growth, MCF‐10A, MCF‐7 and MDA‐MB‐231 cells were seeded at 5 × 10^3^/cm^2^ and 1 × 10^5^/cm^2^, respectively, and cell number was counted every day. As shown in Figure 1A, compared with the cells seeded at 5 × 10^3^/cm^2^, proliferation ability of cells seeded at 1 × 10^5^/cm^2^ was stagnant on the second day (MCF‐10A and MCF‐7) or third day (MDA‐MB‐231). In addition, when cells seeded at 5 × 10^3^/cm^2^, the average values of cell number on second day were 2.01 × 10^4^/cm^2^ (MCF‐10A), 2.27 × 10^4^/cm^2^ (MCF‐7) and 0.82 × 10^4^/cm^2^ (MDA‐MB‐231), and neither of them have reached at high confluent density on fourth day. Based on the results, we chose seeding start at 2 × 10^4^/cm^2^ as normal‐density cells (non‐contact‐inhibited cells), and seeding at 1 × 10^5^/cm^2^ (MCF‐10A and MCF‐7) or 1.5 × 10^5^/cm^2^ (MDA‐MB‐231) as high‐density cells (contact‐inhibited cells). Both normal‐ and high‐density cells were cultured for 2 days. EdU incorporation assay indicated that high‐density cells had a dramatically lower proliferative index (Figure 1B). Typically, activated EGFR signal pathway plays the important roles in cell proliferation, differentiation and others.^20^ Merlin, a tumour suppressor, also regulates proliferation in many cell types.^21^ Next, we detected the phosphorylation levels of EGFR, ERK1/2 (p44/p42) and Merlin in normal‐ and high‐density cells. The results showed that high‐density cells had a striking reduced level of EGFR and ERK1/2 phosphorylation. High‐density cells also showed the decreased phosphorylation at Ser518 of Merlin, potentially indicating the suppression of cell growth (Figure 1C).

### 3.2 Exogenous GM1 promoted contact inhibition of growth in high‐density cells

**Figure 2:**
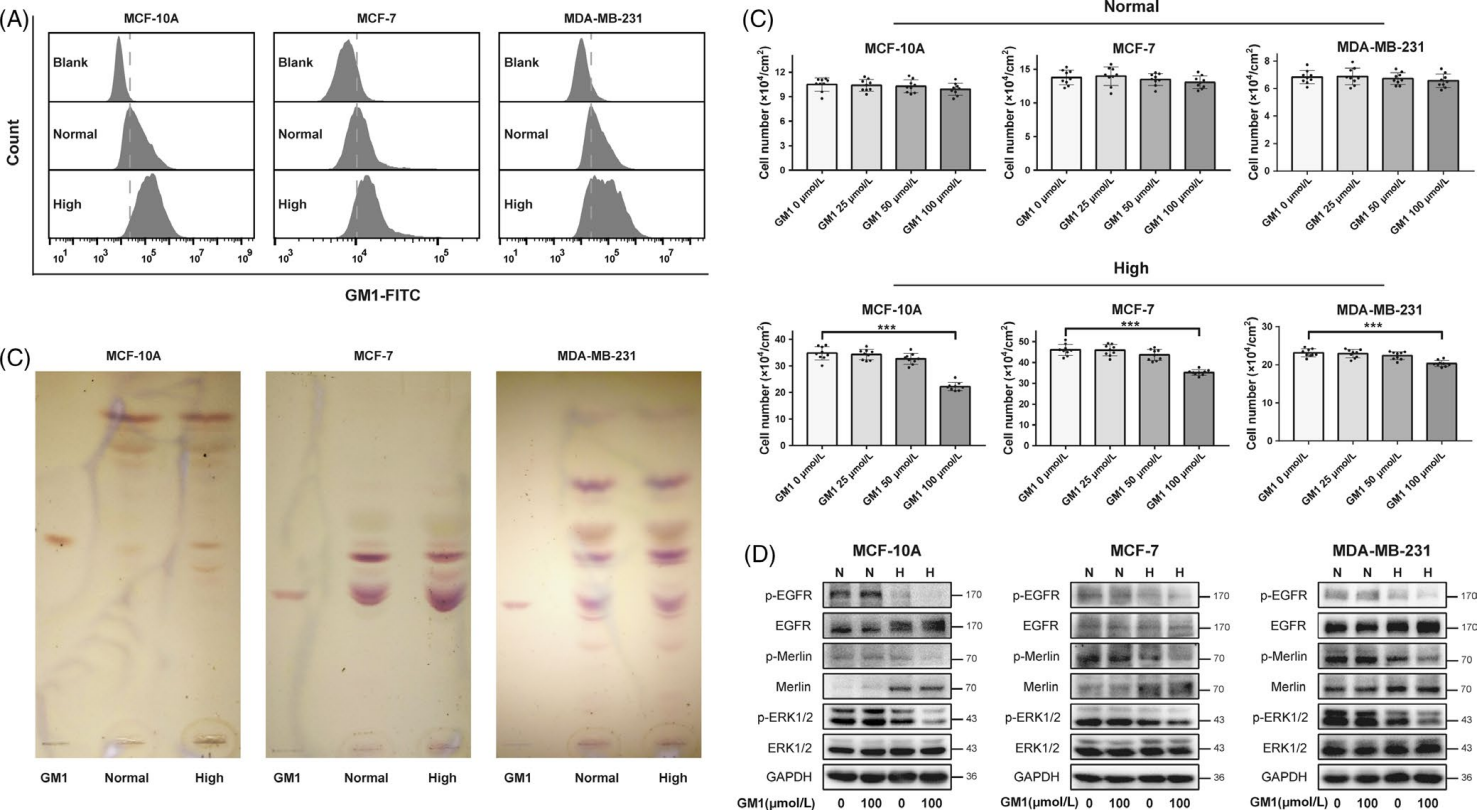
Effect of exogenous addition of GM1 on cell growth. A, Normal‐ and high‐density cells were prepared as described in Figure 1. GM1 expressed on cell surface in normal‐ and high‐density cells was analysed by flow cytometry. B, Normal‐ and high‐density cells were harvested and extracted with isopropanol/hexane/water (55:25:20). GSLs were prepared as described in MATERIALS AND METHODS. After dissolving in chloroform/methanol (2:1), GSLs were spotted on an HPTLC plate, developed with chloroform/methanol/water (55:40:10) and visualized with orcinol/sulphuric acid. C, GM1 was dissolved in serum‐free medium and sonicated for 3 h in sonication bath. Cells were seeded at normal and high density, cultured in complete medium overnight and incubated with 0, 25, 50 and 100 μmol/L GM1 in complete medium (without cholera toxin) for 36 h; cell number were counted; and data were presented as mean ± SD (n = 9). ***P < 0.001. D, Cells were seeded at normal (N) and high (H) density and treated with 100 μmol/L GM1 and culture for 36 h. Phosphorylation levels of EGFR, ERK1/2 and Merlin were analysed by western blotting. GAPDH was used as loading control

In order to study the function of GM1 on contact inhibition of cell growth, we first compared the GM1 expression in normal‐ and high‐density cells by flow cytometry. As shown in Figure 2A, GM1 expression in high‐density was significantly higher than in normal density of MCF‐10A, MCF‐7 and MDA‐MB‐231 cells. HPTLC results showed the same pattern of GM1 expression in normal‐ and high‐density cells (Figure 2B). Next, different concentration of GM1 treatment on both normal‐ and high‐density cells was explored. With the same treatment, exogenous GM1 had no effect on proliferation of normal‐density cells, but exogenous addition of 100 μmol/L GM1 notably inhibited the growth in high‐density cells (Figure 2C). Consistently, phosphorylation of EGFR, ERK1/2 and Merlin was significantly reduced in GM1‐treated high‐density cells (Figure 2D). However, no changes in cell proliferation and phosphorylation of EGFR, ERK1/2 and Merlin were observed in GM1‐treated normal‐density cells. These results illustrated that exogenous addition of GM1 to high‐density cells promoted contact inhibition of growth.

### 3.3 Exogenous GM1 inhibited activation of EGFR signalling in high‐density cells

**Figure 3:**
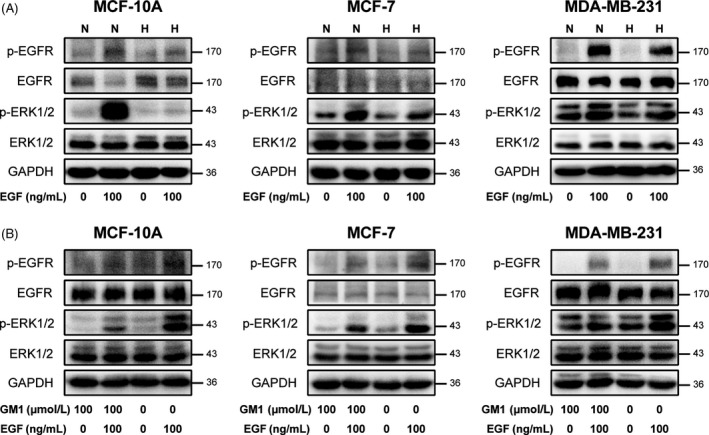
Exogenous GM1 inhibited the activation of EGFR signalling in high‐density cells. A, MCF‐10A, MCF‐7 and MDA‐MB‐231 cells were seeded at normal (N) and high (H) density and cultured for 2 d. Cells were stimulated with 100 ng/mL EGF for 10 min after 8 h serum starvation. B, Cells were seeded at high density, incubation with GM1 for 36 h and then stimulated with EGF. Cells were lysed and subjected to SDS‐PAGE. Phosphorylations of EGFR and ERK1/2 were analysed by western blot as described above

Based on above observations, we speculated GM1 could inhibit the activation of EGFR signalling similarly. Firstly, we activated EGFR pathway by adding EGF to normal‐ and high‐density cells without GM1 incubation. As expected, EGFR and ERK1/2 phosphorylation were increased in both normal‐ and high‐density cells after cells were treated with EGF. However, the phosphorylation levels in high‐density cells were obviously lower than in normal‐density cells after stimulated by EGF (Figure 3A), which demonstrated the activation of EGFR signalling was inhibited in high‐density cells. Next, GM1 was added to high‐density cells before EGF treatment. Results indicated that the activation of EGFR and ERK1/2 was clearly decreased in GM1 treatment cells, compared with no‐treatment group (Figure 3B). These results revealed that the activation of EGFR signalling in high‐density cells was inhibited by exogenous addition of GM1.

### 3.4 EGFR is concentrated in plasma membrane GEM domain of normal‐density cells and translocated to caveolae domain in high‐density cells

**Figure 4:**
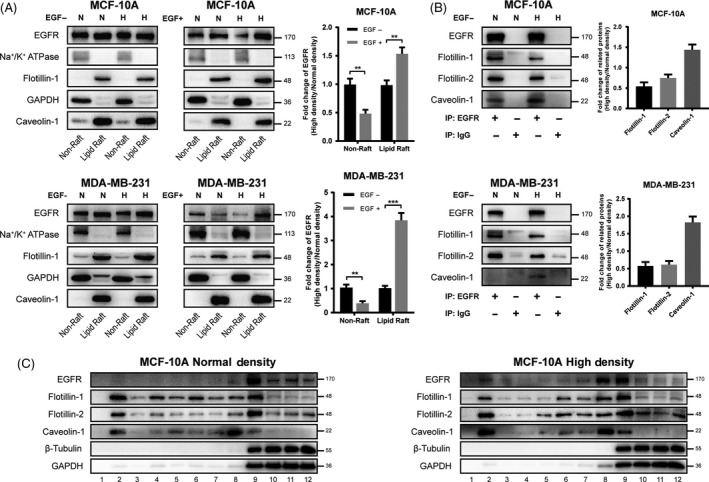
Alteration of EGFR distribution in normal‐ and high‐ density cells. A, MCF‐10A and MDA‐MB‐231 cells were stimulated with or without EGF after serum starvation. Lipid raft and non‐raft fractions were isolated and analysed by western blot. Lipid raft was revealed using anti‐flotillin‐1 and anti‐caveolin‐1 antibodies, and non‐raft part was indicated using anti‐Na^+^/K^+^ ATPase and anti‐GAPDH antibodies. The intensities of band were analysed by using the Image J software. The relative content of EGFR in non‐raft or lipid raft was calculated and shown at the ratio of high density to normal density. **P < 0.01, ***P < 0.001. B, MCF‐10A and MDA‐MB‐231 cells were lysed and immunoprecipitated with anti‐EGFR. EGFR, GEM protein flotillin‐1 and flotillin‐2, and caveolae protein caveolin‐1 were analysed by western blot. The intensities of band were analysed by using the Image J software. The relative content of flotillin‐1, flotillin‐2 and caveolin‐1 was calculated and shown at the ratio of high density to normal density. C, After MCF‐10A cells were cultured for 2 d and starved for 8 h, membrane proteins were separated by density gradient centrifugation. Twelve fractions were collected successively and subjected to western blot analysis. Distribution of EGFR, GEM proteins flotillin‐1 and flotillin‐2, caveolae protein caveolin‐1, non‐raft proteins β‐tubulin and GAPDH was detected by specific antibodies

Usually, EGFR phosphorylation results in receptor internalization and related intracellular signalling.^22^ Hereby, we further monitored the internalization of EGFR in normal‐ and high‐density cells. MCF‐10A and MDA‐MB‐231 cells in normal and high density were starved, and lipid raft and non‐raft fractions were separated. EGFR distribution showed no clear difference between normal‐ and high‐density cells. However, when cells were stimulated with EGF, EGFR content of the high‐density cells showed obvious retention in lipid raft area compared with normal‐density cells (Figure 4A). Next, we examined the different localization of EGFR in GEM and caveolae. In high‐density cells, distribution of EGFR was decreased in GEM, which was recognized by GEM marker flotillin,^23^ but more concentrated in caveolae (Figure 4B). Furthermore, we isolated GEM and caveolae of MCF‐10A cells using detergent‐free OptiPrep gradient method. In both normal‐ and high‐density cells, cytoplasmic protein β‐tubulin and GAPDH were found in fractions 9 ~ 12, while lipid raft maker proteins flotillin and caveolin were mostly presented in fractions 2 ~ 9 (Figure 4C), which are the major fractions of lipid raft. Furthermore, flotillin‐1 and flotillin‐2 were highly enriched in fraction 9, and caveolin‐1 was more presented in fraction 8 rather than fraction nine (Figure 4C), suggesting that GEM was mainly distributed in fraction 8 and caveolae was mainly distributed in fraction 9. EGFR were mainly located in fraction 9 in normal‐density cells, but a portion of EGFR was translocated to fraction 8 in high‐density cells (Figure 4C). These results confirmed that EGFR partially moved from GEM to caveolae in high‐density cells.

### 3.5 GM1 regulates the localization of EGFR outside of GEM domain in high‐density cells

**Figure 5:**
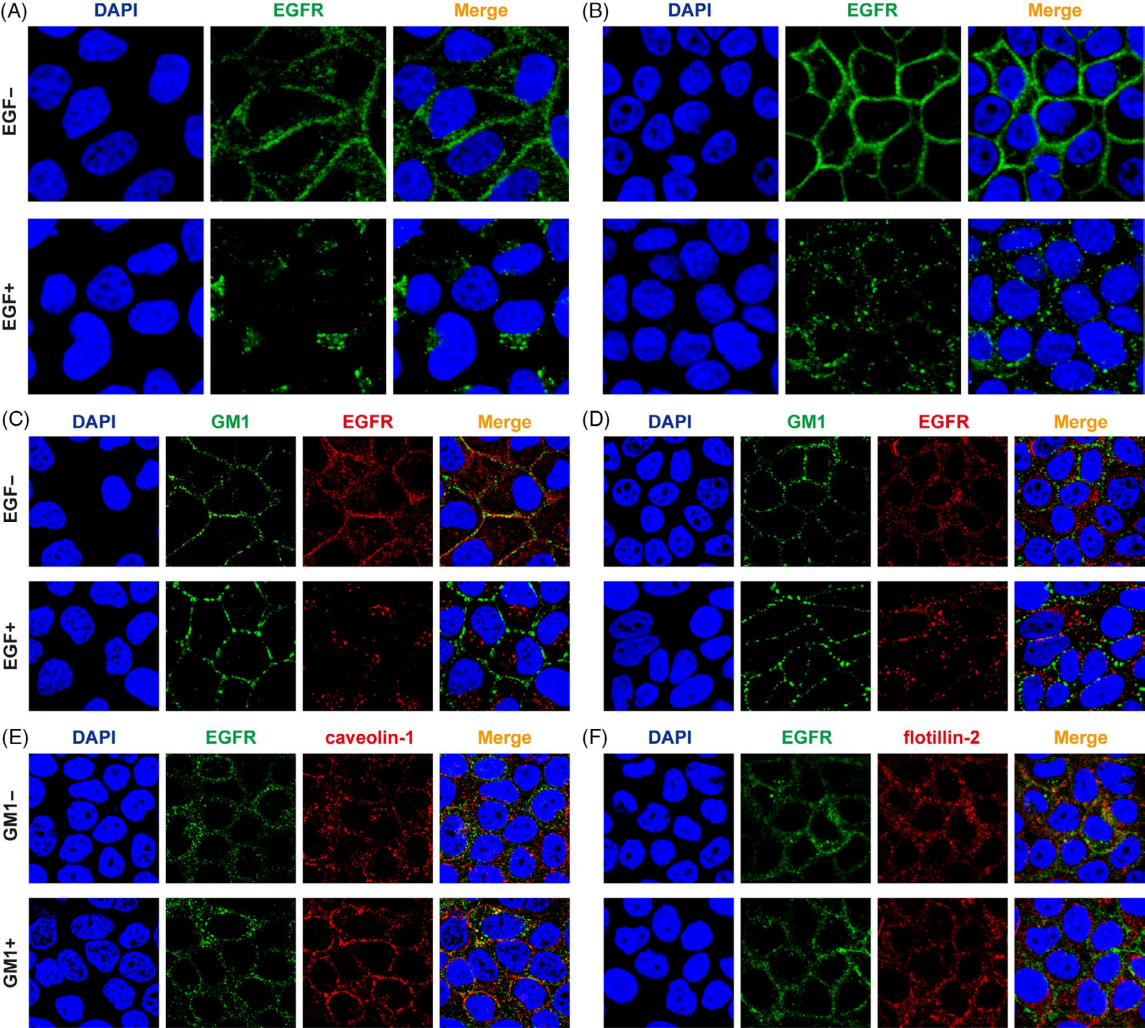
GM1 modulated EGFR localization in high‐density MCF‐10A cells. MCF‐10A cells were seeded at normal density (A, C) and high density (B, D), incubated with GM1 (C, D) or without GM1 (A, B) for 36 h. After cells were starved for 8 h and treated with EGF for 10 min, localization of EGFR was analysed by Immunofluorescence staining. (E, F) Fluorescence distribution of EGFR and caveolin‐1 (E) or flotillin‐2 (F) in high‐density cells treated with GM1 incubation and serum starvation. Magnification, ×600

It has been shown that the activation of EGFR signalling was affected by the distribution of EGFR in two types of lipid rafts: GM1‐enrichment domain and caveolae domain.^24^ We further confirmed the effect of GM1 on EGFR distribution in microdomain by confocal image. The results showed EGFR was internalized into the cytoplasm after EGF stimulation in normal‐density cells (Figure 5A). On the contrary, internalization of EGFR was observably decreased in high‐density cells (Figure 5B). When cells were incubated with GM1 and treated with serum starvation, EGFR was partially found to co‐localize with GM1 in normal‐density cells (Figure 5C), but not in high‐density cells (Figure 5D). Moreover, internalization of EGFR after EGF stimulation was nearly unchanged in normal‐density cells, but was reduced in high‐density cells. Next, we detected the correlation of EGFR with caveolin‐1 and flotillin‐2 under GM1 incubation. In high‐density cells under serum starvation condition, few EGFRs were associated with caveolin‐1 (Figure 5E) and more were located in GEM with flotillin‐2 (Figure 5F). However, when cells were incubated with GM1, EGFR distribution in caveolae was clearly increased in high‐density cells. Taken together, these results showed that GM1 had no discernable impact on EGFR internalization with EGF treatment in normal‐density cells, but dramatically changed the localization of EGFR in high‐density cells and thereby inhibited the activation of EGFR.

### 3.6 GM1 expression alters cell proliferation and EGFR signalling in high‐density cells

**Figure 6:**
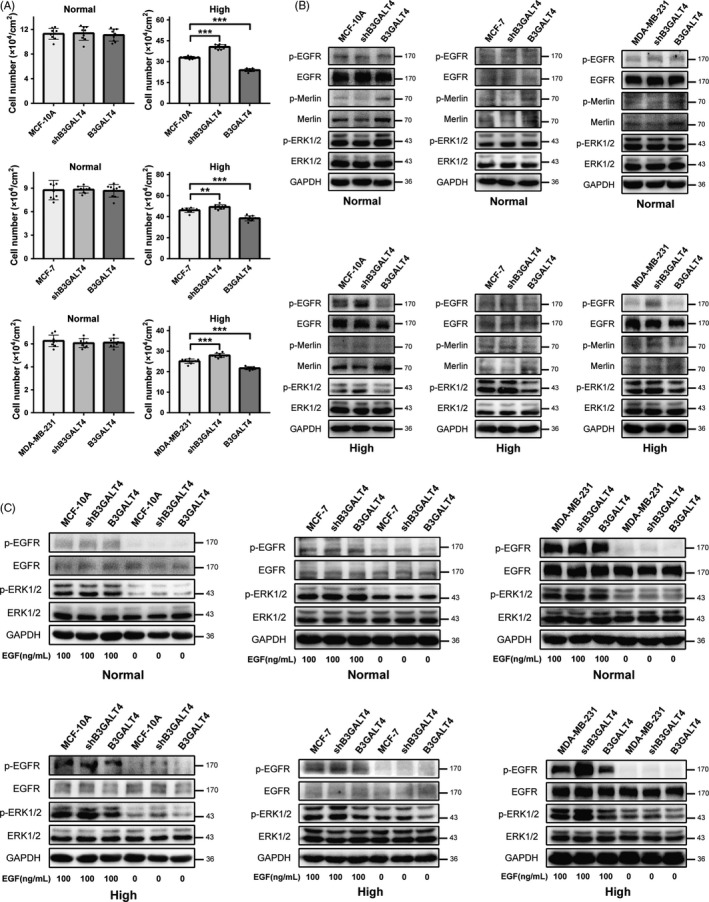
Cell proliferation and EGFR activation of GM1 knockdown and overexpression cells in normal and high‐density culture. A, B, MCF‐10A, MCF‐7, MDA‐MB‐231 and their transfected cells were seeded at normal and high density and cultured for 2 d. Cell number was counted and presented as mean ± SD (n = 9). **P < 0.01, ***P < 0.001 (A). Phosphorylation levels of EGFR, ERK1/2 and Merlin were analysed by western blotting (B). C, Cells were seeded at normal and high density, cultured for 2 d and starved for 8 h. After stimulating with EGF for 10 min, cells were lysed and subjected to SDS‐PAGE. Phosphorylation level of EGFR and ERK1/2 was analysed by western blot

To further confirm GM1 was involved in cell proliferation and EGFR signalling in high‐density cells, B3GALT4 (GM1 synthase gene) was knocked down or overexpressed in MCF‐10A, MCF‐7 and MDA‐MB‐231 cells. As shown in Figure S1, the GM1 expression level was markedly decreased in B3GALT4 knockdown cells and observably increased in B3GALT4 overexpression cells. There were no significant changes of cell proliferation in both wild‐type cells and transfected cells when cultured in normal density. However, in high‐density condition, GM1 knockdown cells exhibited higher proliferative ability and GM1 overexpression cells showed lower proliferative ability compared with wild‐type cells (Figure 6A). Consistent with altered proliferative ability, in normal‐density cells, EGFR, ERK1/2 and Merlin phosphorylation has no clear changes in both wild‐type cells and transfected cells. In high‐density cells, the phosphorylation levels of EGFR, ERK1/2 and Merlin were enhanced in GM1 knockdown cells, but that was reduced in GM1 overexpression cells, compared with wild‐type cells (Figure 6B). Next, we detected the activation of EGFR signalling induced by EGF in GM1 knockdown and overexpression cells. As shown in Figure 6C, in normal‐density cells, there were no obvious differences in the EGFR and ERK1/2 phosphorylation levels in both wild‐type cells and transfected cells after stimulated by EGF. By contrast, when treated with EGF in high‐density cells, phosphorylation of EGFR and ERK1/2 was higher in GM1 knockdown cells compared with wild‐type cells and that was lower in GM1 overexpression cells compared with wild‐type cells. These results further corroborated that GM1 could modulate contact inhibition of growth in human mammary epithelial cells.

## 4 DISCUSSION

Density‐dependent inhibition of cell growth has been recognized in the 1950s,^25^ and the concept of “contact inhibition” has been formally proposed in the 1960s.^8^ Subsequent studies have found that contact inhibition plays an important role in modulation of tissue growth, differentiation and development.^26^ Several signalling pathways have been implicated in regulation of contact inhibition,^27^ and one of the most popular and studied pathways is Hippo pathway.^28^ The neurofibromatosis type 2 (NF2) tumour suppressor, Merlin, is identified as an upstream regulator of this pathway.^29^ Merlin can negatively regulate EGFR signalling by retaining EGFR into a membrane compartment preventing it signalling and internalization.^30^ EGFR is one of the receptor tyrosine kinase families, and EGFR affinity to EGF is specifically decreased in high‐density cells without the change in total receptor number.^31^ EGFR activity can be modulated by specific gangliosides, and some gangliosides can directly bind to EGFR result in inhibition of EGFR activation.^32^ However, the interaction between GM1 and EGFR, and the function of GM1 in contact inhibition have been reported rarely. Thus, it is necessary to further characterize connection between GM1 and EGFR, and identification of the role of GM1 in contact inhibition will serve to emphasize the roles of GSLs in many essential biological processes such as cell growth.

In present study, we detected the existence of contact inhibition of growth in high density of MCF‐10A, MCF‐7 and MDA‐MB‐231 cells by using cell growth curve, EdU incorporation assays and western blot. Previous research showed that qualitative and quantitative composition of GSLs in human skin fibroblasts is specifically changed depending on cell density.^33^ Our results found that the expression of GM1 was increased in high‐density cells, and exogenous addition of GM1 to high‐density cells could clearly promote the contact inhibition of growth. Also, phosphorylation levels of EGFR, ERK1/2 and Merlin were obviously reduced in GM1‐treated high‐density cells. Furthermore, western blot analysis of serum‐starved cells that treated with EGF revealed that the activation of EGFR signalling in high‐density cells was inhibited by exogenous addition of GM1.

Our previous studies showed GM3 could inhibit EGFR activation by directly binding to GlcNAc of N‐Linked glycan of EGFR.^34^ In this study, we also confirmed that GM3 could inhibit cell proliferation and EGFR activation in both normal‐ and high‐density cells (Figure S2). By contrast, GM1 has a weak capacity to inhibit human neuroblastoma cell proliferation and EGFR phosphorylation.^35^ However, in mouse fibroblast cells, treatment with GM1 could markedly reduce phosphorylation of PDGFR by excluding the PDGFR from GEM domain.^36^ Overexpressed GM1 suppresses the TrkA activation by regulating the distribution of receptor from lipid raft fraction to the non‐raft fraction in PC12 cells.^37^ Here, we found that in GM1 pre‐treated cells, blockage GM1 with CTB cannot restore the cell proliferation ability (Figure S3). The distribution and activation of EGFR are mainly in lipid raft domain, but not in caveolae,^14^, ^15^, ^38^ and caveolin could bind to EGFR and inhibit the activation of EGFR.^39^, ^40^ Therefore, we speculated that GM1 may inhibit growth of high‐density cells by changing the distribution of EGFR in lipid raft. Firstly, we detected the distribution of EGFR in lipid raft and no‐raft, and found that after EGF treated, EGFR content of high‐density cells was obviously retarded in the lipid raft area compared with normal‐density cells. Co‐IP analysis and OptiPrep gradient method indicated that in high‐density cells, distribution of EGFR was decreased in GEM, but more concentrated in caveolae. Immunofluorescence staining showed that GM1 dramatically changed the localization of EGFR in high‐density cells and thereby inhibited the activation of EGFR. In addition, the cell proliferation and EGFR activation were increased in GM1 knockdown cells and decreased in GM1 overexpression cells when cultured in high density.

**Figure 7:**
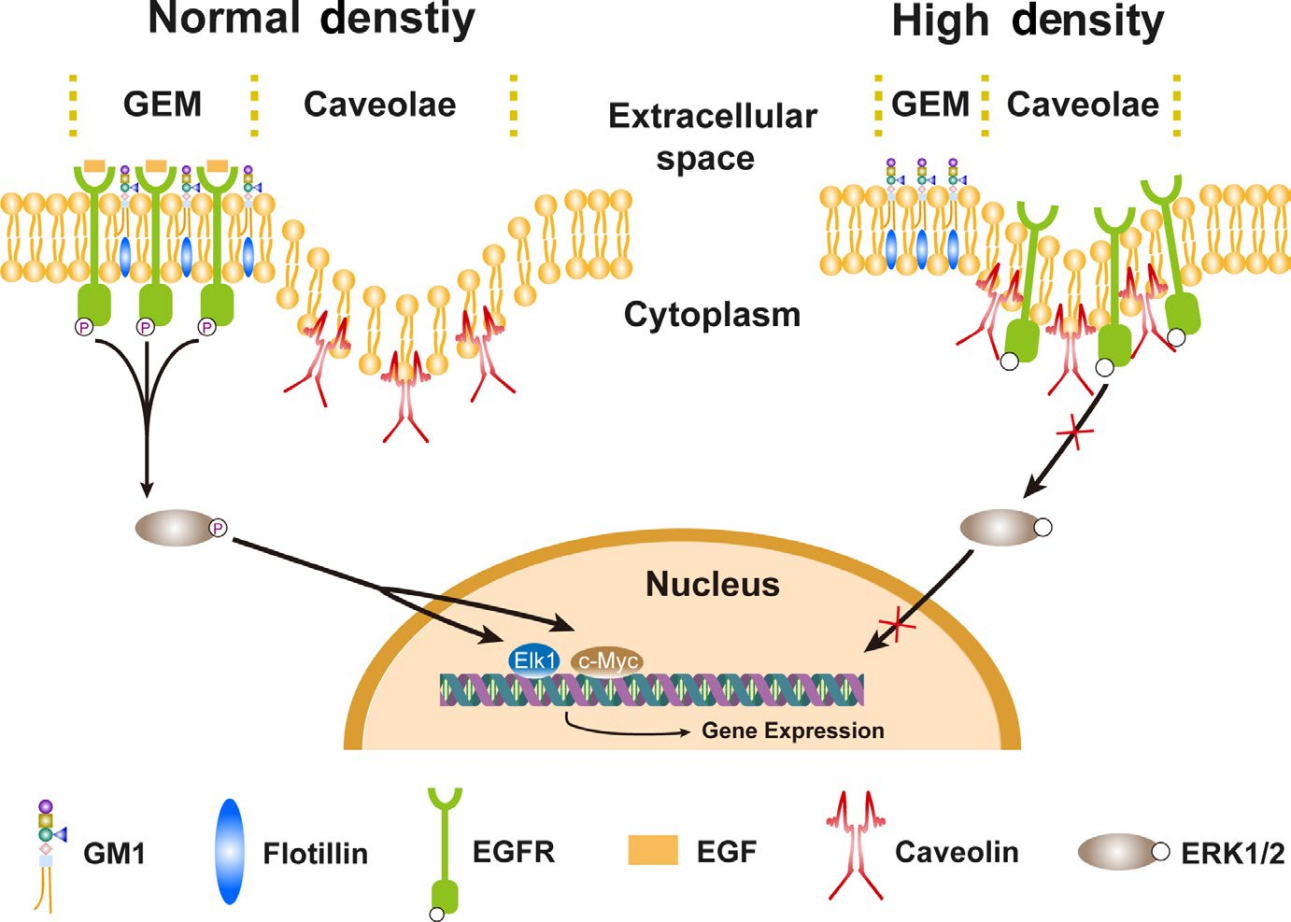
The schematic model of GM1 inhibiting EGFR activation and promoting contact inhibition of growth through regulating the distribution of EGFR from GEM domain to caveolae domain

In conclusion, we identified that in MCF‐10A, MCF‐7 and MDA‐MB‐231 human mammary epithelial cells, expression of GM1 was increased in contact‐inhibited cells, and exogenous addition of GM1 or overexpression of GM1 inhibited cell proliferation and EGFR activation in high‐density cells. Despite many details are still unknown, we put forward an assumption that GM1 suppresses EGFR activation by changing the localization of EGFR from GEM domain to caveolae domain and further promotes contact inhibition of growth (Figure 7). The detailed molecular mechanism needs to be explored in the future studies.

## CONFLICTS OF INTEREST

The authors in this research disclose no conflicts.
