# Corrigendum: Ganglioside GM1 promotes contact inhibition of growth by regulating the localization of epidermal growth factor receptor from glycosphingolipid‐enriched microdomain to caveolae Article DOI: 10.1111/cpr.12639

**DOI:** 10.1111/cpr.12748

**Published:** 2020-01-27

**Authors:** Dinghao Zhuo, Feng Guan

In the published version of this article, author Guan Feng failed to include “Key Laboratory of Carbohydrate Chemistry and Biotechnology, Ministry of Education, School of Biotechnology, Jiangnan University, Wuxi, China,” as his first organization in affiliation section.

The original text and the modified text are shown below:

The original version:

Ganglioside GM1 promotes contact inhibition of growth by regulating the localization of epidermal growth factor receptor from glycosphingolipid‐enriched microdomain to caveolae

Zhuo Dinghao^1^, Guan Feng^2,*^

^1^Key Laboratory of Carbohydrate Chemistry and Biotechnology, Ministry of Education, School of Biotechnology, Jiangnan University, Wuxi, China.

^2^Provincial Key Laboratory of Biotechnology, Joint International Research Laboratory of Glycobiology and Medicinal Chemistry, College of Life Science, Northwest University, Xi'an, China.

The modified version:

Ganglioside GM1 promotes contact inhibition of growth by regulating the localization of epidermal growth factor receptor from glycosphingolipid‐enriched microdomain to caveolae

Zhuo Dinghao^1^, Guan Feng^1,2,*^

^1^Key Laboratory of Carbohydrate Chemistry and Biotechnology, Ministry of Education, School of Biotechnology, Jiangnan University, Wuxi, China.

^2^Provincial Key Laboratory of Biotechnology, Joint International Research Laboratory of Glycobiology and Medicinal Chemistry, College of Life Science, Northwest University, Xi'an, China.

The author would like to sincerely apologize for the inconvenience.
