# Supplementary material for: Ganglioside GM1 promotes contact inhibition of growth by regulating the localization of epidermal growth factor receptor from glycosphingolipid‐enriched microdomain to caveolae
Source: Cell Prolif. 2019 May 24;52(4):e12639. doi: 10.1111/cpr.12639 (PMC6668969; doi:10.1111/cpr.12639)
Supplement: Supplementary file 1 [file CPR-52-e12639-s001.docx]

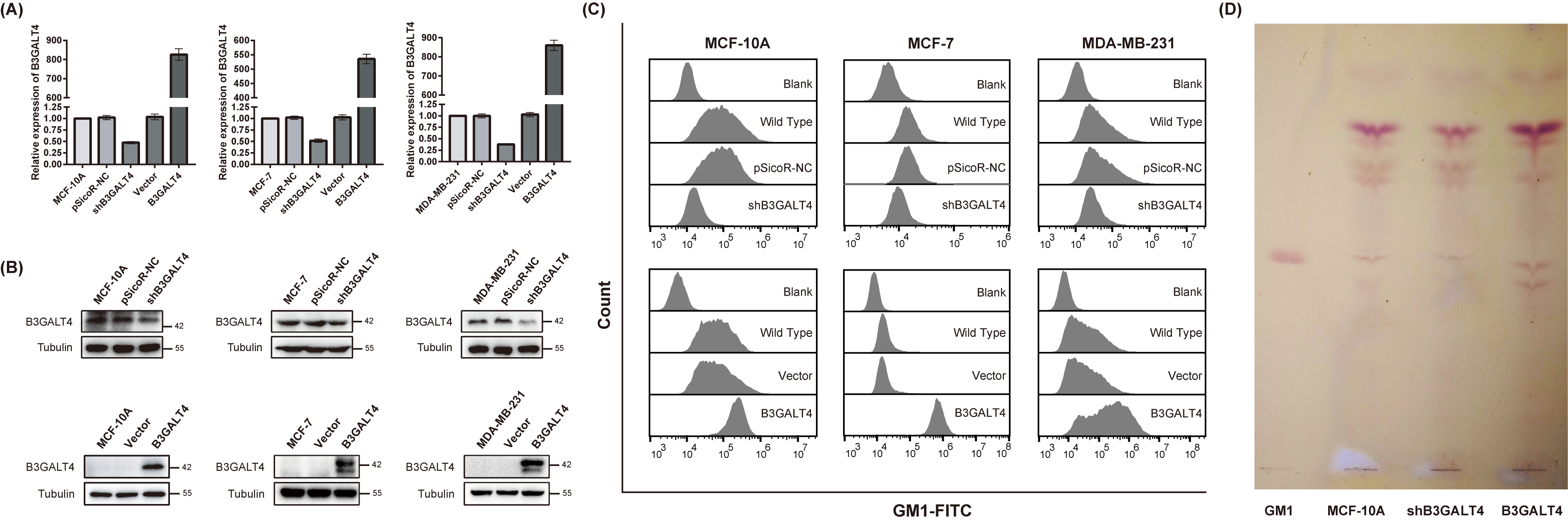


**FIGURE S1** Construction of GM1 knockdown and overexpression cells. A, mRNA expression levels of *B3GALT4* in transfected cells were determined by Quantitative real-time RT-PCR. Values were presented as mean ± SD (n= 3). B, protein expression levels of B3GALT4 were analyzed by western blot. (C, D) GM1 expression levels were analyzed by Flow cytometric (C) and HPTLC (D).


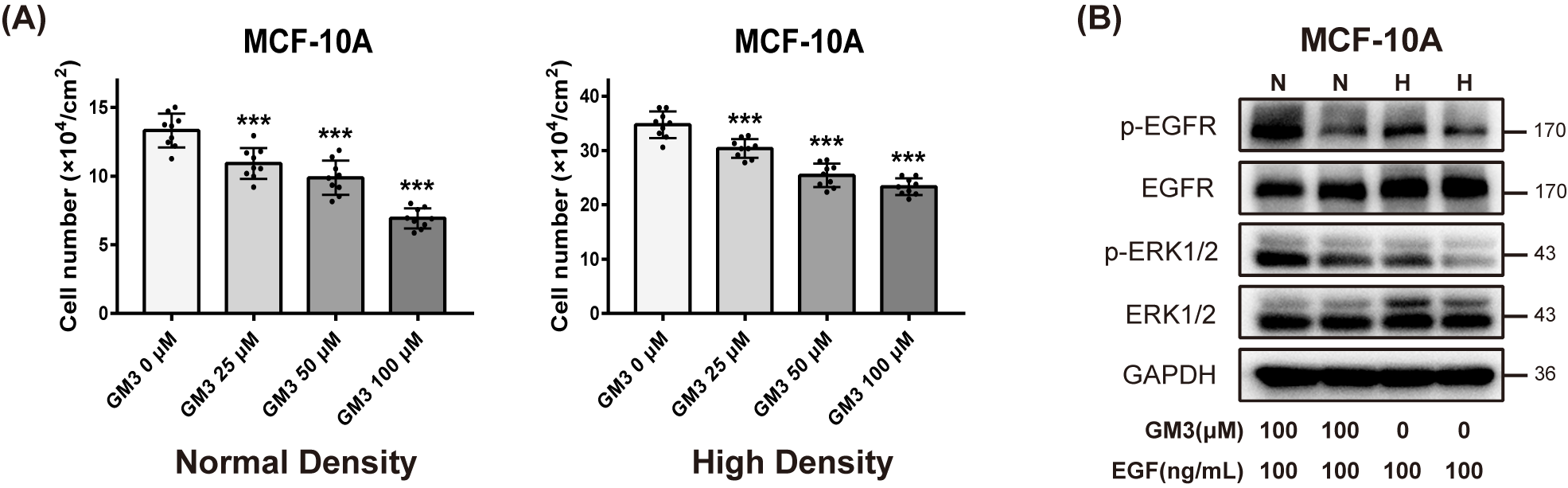


**FIGURE S2** GM3 inhibit cell proliferation and EGFR activation. A, MCF-10A cells were seeded and treated GM3 for 36 h, and cell number were counted, and data were presented as mean ± SD (n = 9). ***p< 0.001. B, MCF-10A cells were seeded at normal (N) and high (H) density, treated with GM3, stimulated with EGF. Phosphorylation levels of EGFR and ERK1/2 were analyzed by western blotting.


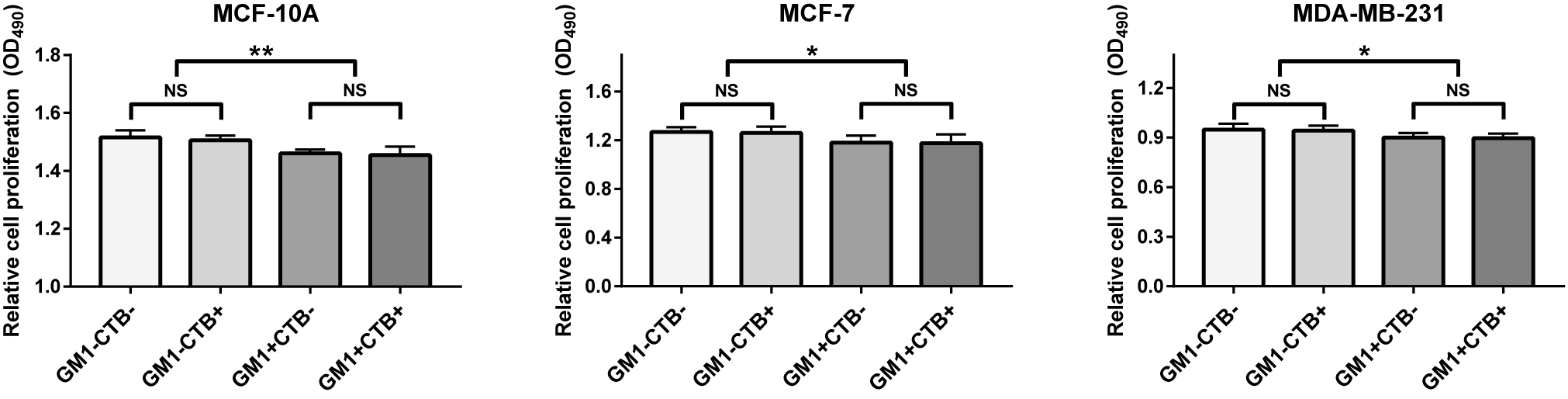


**FIGURE S3** GM1 blockade can’t restore the proliferation ability. MCF-10A, MCF-7 and MDA-MB-231 cells were seeded at normal density and cultured in complete mediums overnight. Mediums were changed to complete mediums or conditioned mediums containing GM1 and culture was continued for 36 hr. Cells were then incubated with 2.5 ug/ mL of CTB and further cultured for 2 days. Cell proliferation ability was then detected through MTS assay. Data were presented as mean ± SD (n=5) *p< 0.05, **p< 0.01.
